# ChrXq27.3 miRNA cluster functions in cancer development

**DOI:** 10.1186/s13046-021-01910-0

**Published:** 2021-03-25

**Authors:** Kosuke Yoshida, Akira Yokoi, Yusuke Yamamoto, Hiroaki Kajiyama

**Affiliations:** 1 Department of Obstetrics and Gynecology, Nagoya University Graduate School of Medicine, Tsuruma-cho 65, Showa-ku, Nagoya, 466-8550 Japan; 2 Institute for Advanced Research, Nagoya University, Nagoya, Japan; 3 Division of Cellular Signaling, National Cancer Center Research Institute, Tokyo, Japan

**Keywords:** miRNA, X chromosome, miRNA cluster, Epithelial–mesenchymal transition, Proliferation, Drug-resistance, miR-506-3p, miR-888-5p

## Abstract

MicroRNAs (miRNAs) regulate the expression of their target genes post-transcriptionally; thus, they are deeply involved in fundamental biological processes. miRNA clusters contain two or more miRNA-encoding genes, and these miRNAs are usually coexpressed due to common expression mechanisms. Therefore, miRNA clusters are effective modulators of biological pathways by the members coordinately regulating their multiple target genes, and an miRNA cluster located on the X chromosome q27.3 region has received much attention in cancer research recently. In this review, we discuss the novel findings of the chrXq27.3 miRNA cluster in various types of cancer.

The chrXq27.3 miRNA cluster contains 30 mature miRNAs synthesized from 22 miRNA-encoding genes in an ~ 1.3-Mb region. The expressions of these miRNAs are usually negligible in many normal tissues, with the male reproductive system being an exception. In cancer tissues, each miRNA is dysregulated, compared with in adjacent normal tissues. The miRNA-encoding genes are not uniformly distributed in the region, and they are further divided into two groups (the miR-506-514 and miR-888-892 groups) according to their location on the genome. Most of the miRNAs in the former group are tumor-suppressive miRNAs that are further downregulated in various cancers compared with normal tissues. miR-506-3p in particular is the most well-known miRNA in this cluster, and it has various tumor-suppressive functions associated with the epithelial–mesenchymal transition, proliferation, and drug resistance. Moreover, other miRNAs, such as miR-508-3p and miR-509-3p, have similar tumor-suppressive effects. Hence, the expression of these miRNAs is clinically favorable as prognostic factors in various cancers. However, the functions of the latter group are less understood. In the latter group, miR-888-5p displays oncogenic functions, whereas miR-892b is tumor suppressive. Therefore, the functions of the miR-888–892 group are considered to be cell type- or tissue-specific.

In conclusion, the chrXq27.3 miRNA cluster is a critical regulator of cancer progression, and the miRNAs themselves, their regulatory mechanisms, and their target genes might be promising therapeutic targets.

## Background

MicroRNAs (miRNAs), which are small noncoding RNA molecules (~ 22 nucleotides in length), regulate gene expression by interacting with the 3′-untranslated regions of genes [1, 2]. Numerous studies have revealed the functions of miRNAs in various cancers [2]. Some miRNA-encoding genes are located in narrow regions on the genome, which are so-called miRNA clusters; there are 159 miRNA clusters in the human genome [3]. In an miRNA cluster, miRNA-encoding genes can be under the control of a common regulatory unit and are coexpressed [3, 4]. Moreover, members of an miRNA cluster have the same targets or target different genes belonging to specific pathways [3]. One of the most well-known miRNA clusters is the miR-17–92 cluster, which is located on chromosome 13q31.3. This region is amplified in lung cancer and B-cell lymphomas, and the expression of the miRNAs derived from the miR-17–92 cluster is substantially increased in these conditions [5, 6]. Functionally, the miR-17–92 cluster is considered an oncogene and acts with *c-myc* to promote tumor development [5]. An miRNA cluster located in the chrXq27.3 region (the chrXq27.3 miRNA cluster) has received much attention recently, and the oncogenic or tumor-suppressive functions of this cluster have been elucidated through studies on various cancers.

In this review, we provide an overview of the chrXq27.3 miRNA cluster in cancer progression.

## chrXq27.3 miRNA cluster

**Fig. 1:**
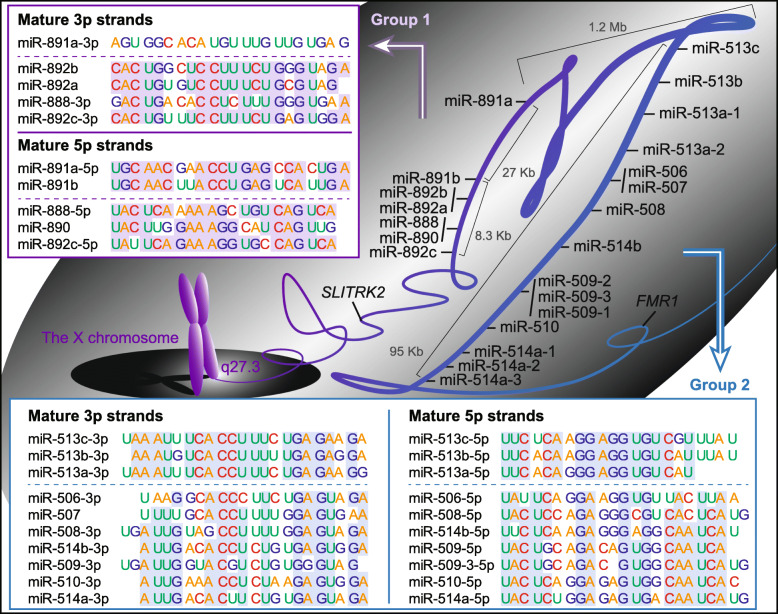
Schema of the chrXq27.3 miRNA cluster and sequence similarity of the miRNAs in the cluster There are 22 miRNA-encoding genes in the chrXq27.3 cluster, and they are divided into two groups according to their location. Mature forms of the miRNAs with close loci tend to have similar sequences

The chrXq27.3 miRNA cluster contains 30 mature miRNAs synthesized from 22 miRNA-encoding genes in an ~ 1.3-Mb region (Fig. 1). The miRNA-encoding genes are not uniformly distributed in the region and are separated by a noncoding region of ~ 1.2-Mb. There are 15 miRNA-encoding genes downstream of the noncoding region, and they are named the miR-506–514 group. Similarly, the rest of seven genes are located on the upstream of the noncoding region, and named the miR-888–892 groups. Evolutionarily, mammalian species have an miRNA cluster located between the *FMR1* and *SLITRK2* genes on the X chromosome, and this miRNA cluster is well conserved among primate species [7–9]. Moreover, mature forms of miRNAs with close loci tend to have similar sequences, and hence, these miRNAs can have common target genes [7, 8].

Many miRNA genes are located inside or close to fragile sites. The chrXq27.3 region is well-known as a key spot for fragile-X syndrome, the most common form of hereditary intellectual disability. This syndrome is responsible for increased CGG repeats in the *FMR1* gene [10]. However, it remains unclear whether the chrXq27.3 cluster is involved in this syndrome even though some of the miRNAs can target the adjacent gene, *FMR1* [9]. Conversely, the expression of these miRNAs is usually negligible in normal tissues except for in the male reproductive system [8, 9, 11]. Thus, this cluster is thought to be involved in testis development and spermatogenesis, and its alteration might be associated with male infertility [8, 9, 11, 12]. Additionally, according to Pinheiro’s hypothesis, X-linked miRNAs might contribute to the immunological advantage of females as the X chromosome contains 10% of all human miRNAs whereas the Y chromosome has no miRNAs, and several X-linked miRNAs have important functions in immunity and cancer [13]. Therefore, the chrXq27.3 miRNA cluster may be important from the aspect of gender differences.

## Clinical significance of the chrXq27.3 miRNA cluster in various cancers

In cancer research, each miRNA is reported to be dysregulated in various cancer tissues compared with in adjacent normal tissues. The most well-known miRNA in the cluster is miR-506-3p, which is commonly downregulated regardless of the type of cancers, including gastric, colorectal, pancreatic, hepatocellular, lung, breast, ovarian, uterine cervical, renal, bladder, nasopharyngeal, and thyroid carcinoma [14–25]. Moreover, as cancer progresses, miR-506-3p expression further decreases, and decreased miR-506-3p expression is associated with poor prognosis in patients with these cancers [16, 18, 22, 26–28] (Table 1). Similarly, the rest of the miR-506–514 group is also tumor-suppressive, and they are frequently downregulated in various cancer tissues compared with normal tissues [29–47]. miR-508-3p and miR-509-3p are further downregulated in cancer cells with treatment resistance; thus, this downregulation is associated with poor survival [48, 49]. Moreover, in breast cancer, low miR-507 and miR-508-3p expression are associated with distant and lymph node metastases, and miR-509-5p and miR-509-3-5p are remarkably decreased in brain metastases compared with primary cancer [31, 50, 51]. Furthermore, several miRNAs in the cluster, including miR-508-3p, miR-509-3p, miR-509-3-5p, and miR-514a-3p, are frequently decreased in advanced and recurrent ovarian carcinoma [52, 53]. Hence, these miRNAs are considered to be coregulated and coexpressed. Other reports have also noted that the expression of these miRNAs is associated with poor prognosis in patients with various cancers [30, 48, 49, 52, 54–56]. However, there are some exceptions. For example, high miR-508-3p expression is associated with shorter disease-free and overall survivals in esophageal squamous cell carcinoma (SCC) [57]. Moreover, high miR-513a-5p expression in breast cancer is a poor prognostic factor [58]. Furthermore, the upregulation of miR-509-5p, miR-510-3p, and miR-510-5p has been reported in thyroid and lung cancer, although their prognostic impacts have not been investigated [59–62]. Therefore, the miR-506–514 group, which act as tumor suppressors, is usually downregulated in cancer tissues compared with normal tissues.

**Table 1:** Prognostic impact of the chrXq27.3 miRNA cluster

	Esophageal	Gastric	Colorectal	Pancreatic	Hepatocellular	Lung	Breast	Ovarian	Renal
miR-506-3p		Favorable		Favorable		Favorable	Favorable	Favorable	Favorable
miR-507						Favorable			
miR-508-3p	Poor		Favorable					Favorable	
miR-508-5p		Favorable							
miR-509-3p								Favorable	
miR-509-5p				Favorable					
miR-513a-5p							Poor		
miR-888-5p			Poor						
miR-891b				Favorable					
miR-892a					Poor				
miR-892b							Favorable		

Favorable, the expression is associated with favorable prognosis; Poor, the expression is associated with poor prognosis

Conversely, the miR-888–892 group has both oncogenic and tumor-suppressive functions depending on the miRNAs. miR-888-5p and miR-892a are upregulated in colorectal and hepatocellular carcinoma, and higher miR-888-5p or miR-892a expressions are associated with poor survival (Table 1) [63–66]. However, miR-890 and miR-892b are downregulated in breast cancer, and lower miR-892b expression is associated with a poor prognosis [67, 68]. Moreover, miR-892b is downregulated in pancreatic and nasopharyngeal cancer tissues, and lower miR-891b expression is associated with shorter survival in pancreatic cancer [69–71].

Therefore, the alteration of the miRNAs in this cluster is frequently observed in various cancers. Moreover, their roles are usually common across cancer types and are considered critical for cancer development.

## The functions of the chrXq27.3 miRNA cluster in cancer progression

The detailed functions of each miRNA in the cluster are described below; however, no reports have yet been published about the following seven miRNAs: miR-513a-3p, miR-513b-3p, miR-513c-3p, miR-888-3p, miR-891a-3p, miR-892c-3p, and miR-892c-5p.

### miR-506-3p, miR-506-5p, and miR-507

**Fig. 2:**
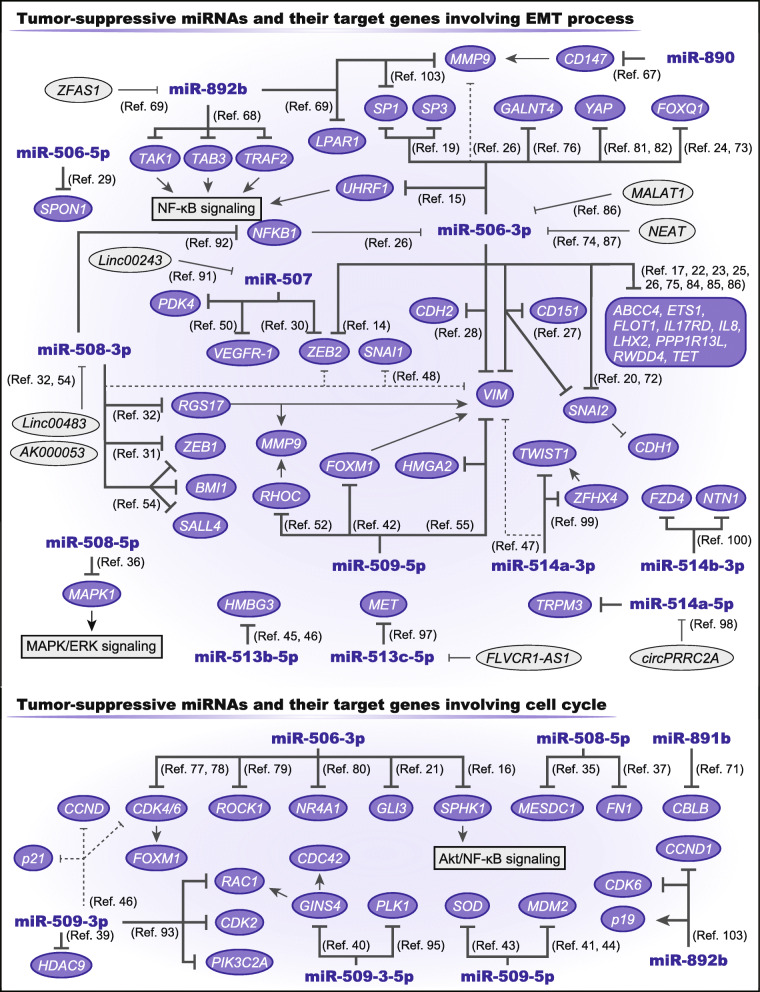
Tumor-suppressive roles of the chrXq27.3 miRNA cluster Many miRNAs in the cluster act as tumor suppressors by targeting various genes involved in the epithelial–mesenchymal transition and proliferation. Previously validated interactions between the miRNAs and genes or non-cording RNAs are described

One of the most important functions of miR-506-3p is regulation of the epithelial–mesenchymal transition (EMT), which is a critical process to gain migratory and invasive potential in cancer development. Consistent with the clinical tumor-suppressive effect described above, miR-506-3p inhibits EMT by directly targeting key genes of the process; including *ZEB2*, *SNAI2*, *VIM*, and *CDH2* (Fig. 2) [14, 20, 27, 28, 72]. Moreover, through regulating other target genes, miR-506-3p induces the downregulation of *VIM*, *SNAIL2*, *TWIST,* and *CDH2* and the upregulation of *CDH1* [17, 25–27, 73–75]. Furthermore, miR-506-3p contributes to maintenance of the tumor microenvironment by decreasing *MMP9* expression and inhibiting angiogenesis [26, 76]. Therefore, miR-506-3p is an important regulator of cancer invasion and metastasis.

**Fig. 3:**
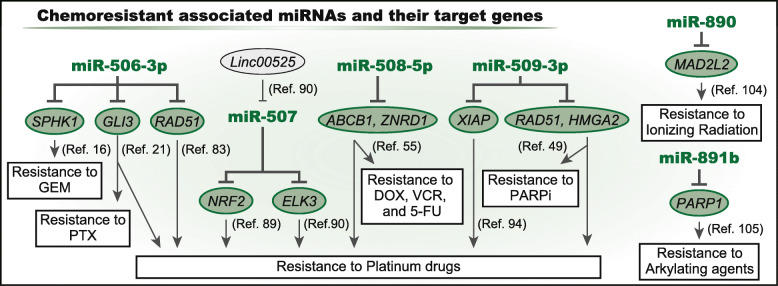
Treatment resistance and the chrXq27.3 miRNA cluster Several miRNAs in the cluster are involved in treatment resistance, and their previously validated functions are described

In addition to inhibiting the EMT, miR-506-3p suppresses proliferation by directly targeting *CDK4/6*. Other target genes such as *GLI3*, *ROCK1*, and *NR4A1* are also involved in miR-506-3p-induced cell cycle arrest [21, 77–80]. Moreover, miR-506-3p modulates the NF-κB and Hippo signaling pathways and is associated with tumor suppression [15, 16, 81, 82]. Thus, miR-506-3p can enhance the efficacy of anti-cancer drugs and can sensitize cancer cells to DNA damage by targeting *RAD51*, *GLI3*, and *SPHK1* (Fig. 3) [16, 21, 83]. Furthermore, many other target genes of miR-506-3p are reported to be associated with cancer progression [19, 22–24, 84, 85]. Additionally, MALAT1 and NEAT1, which are long noncoding RNA (lncRNA), can modulate miR-506-3p expression [74, 86, 87]. Therefore, miR-506-3p is a common tumor suppressor across various cancers, and miR-506-3p-associated genes can be therapeutic targets.

However, the functions of miR-506-5p remain largely unknown. In hepatocellular carcinoma cells, miR-506-5p suppresses proliferation, migration, and invasion by regulating *SPON1* (Fig. 2) [29]. A recent study showed that miR-506-5p also induces the downregulation of *VIM, CDH2*, and *MMP9* and the upregulation of *CDH1* in glioma cells [88]*.* Therefore, miR-506-5p and miR-506-3p might coordinately regulate the EMT.

The miR-507 encoding gene is located only ~ 300 bp downstream of the miR-506 encoding gene, and the mature sequence of miR-507 is similar to that of miR-506-3p. Therefore, miR-507 also directly targets *ZEB2* and *VEGFR-1* and contributes to cell migration and invasion (Fig. 2) [30, 50]. Moreover, miR-507 enhances platinum sensitivity via targeting *NRF2* and *ELK3* (Fig. 3) [89, 90]. Furthermore, miR-507 decreases glucose uptake and lactate production by targeting *PDK4*; thus, lncRNA LINC00243 promotes proliferation and glycolysis by sponging miR-507 [91]. Overall, these miRNAs function as tumor-suppressive miRNAs in variety of cell types.

### miR-508-3p and miR-508-5p

**Fig. 4:**
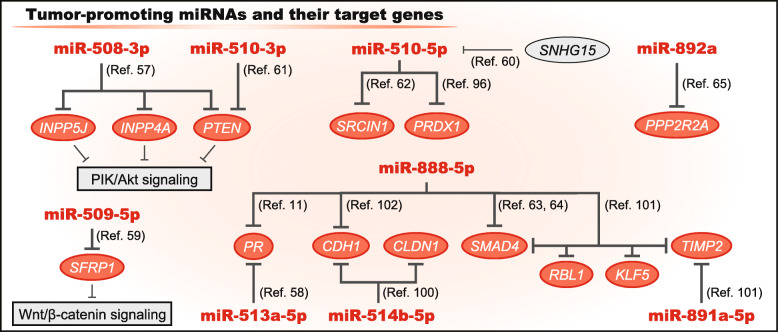
Tumor-promotive roles of the chrXq27.3 miRNA cluster Some miRNAs in the cluster also act in an oncogenic manner by targeting various genes. Previously validated interactions between the miRNAs and genes or non-cording RNAs are described

A network biology analysis revealed that miR-508-3p is strongly associated with mesenchymal properties in ovarian cancer, and it indirectly regulates multiple EMT-associated genes (Fig. 2) [48]. Other reports have highlighted the direct interaction between miR-508-3p and *ZEB1* [31, 54]. Moreover, in addition to the EMT, miR-508-3p regulates stemness by targeting *BMI1* and *SALL4* [54]. Furthermore, *NFKB1* is targeted by miR-508-3p; thus, miR-508-3p downregulation contributes to canonical NF-κB activation [92]. Therefore, miR-508-3p has similar functions as miR-506-3p and is a strong tumor suppressor. Hence, lncRNAs silence the expression of miR-508-3p and exert their oncogenic functions [32, 54]. However, miR-508-3p may display an opposition function in esophageal SCC. A previous report showed that miR-508-3p sustained PI3K/Akt signaling by targeting the tumor suppressive genes *PTEN, INPP5J*, and *INPP4A*, resulting in an increasingly aggressive phenotype of esophageal SCC (Fig. 4) [57].

miR-508-5p targets *MAPK1* and suppresses the EMT by modulating MAPK/ERK signaling (Fig. 2) [36]. Moreover, miR-508-5p attenuates proliferation and invasion by targeting *FN1* and *MESDC1* [35, 37]. In gastric cancer cells, miR-508-5p reverses resistance to doxorubicin, vincristine, 5-fluorouracil, and cisplatin by targeting *ABCB1* and *ZNRD1* (Fig. 3) [55]. Therefore, except for miR-508-3p in esophageal SCC, these miRNAs act as tumor suppressors.

### miR-509-3p, miR-509-5p, and miR-509-3-5p

In a narrow region of ~ 2 Kb, there are three precursors of the miR-509 family: miR-509-1, miR-509-2, and miR-509-3 (Fig. 1). Although their 3p strands are similar, miR-509-1 and miR-509-2 synthesize miR-509-5p whereas miR-509-3 synthesizes miR-509-3-5p.

Similar to the miRNAs previously described, miR-509-3p is a tumor suppressor and exerts tumor-suppressive effects by targeting several critical regulators, including *CDK2*, *RAC1*, and *PIK3C2A* (Fig. 2) [93]. Moreover, miR-509-3p inhibits proliferation and increases apoptosis by targeting *HDAC9* [39]. miR-509-3p also plays a role in treatment resistance by sensitizing ovarian cancer cells to cisplatin and olaparib by targeting *XIAP*, *HMGA2,* and *RAD51* (Fig. 3) [49, 94].

miR-509-5p has similar target genes, *VIM* and *HMGA2* (Fig. 2) [56]. miR-509-5p inhibits EMT and proliferation by targeting *FOXM1*, *MDM2,* and *SOD* [41–44]. In a mouse model, miR-509-5p was shown to protect brain metastasis by targeting two essential genes, *RHOC* and *TNF* [51]. Needless to say, miR-509-3-5p has a very similar sequence to miR-509-5p, and thus they might have common target genes (Fig. 1). miR-509-3-5p suppresses the activation of RAC1 and CDC42 by targeting *GINS4* and induces G2/M arrest by targeting *PLK1* [40, 95]. Thus, these miRNAs are tumor suppressive. However, in papillary thyroid carcinoma, miR-509-5p displays oncogenic functions and promotes activation of the Wnt/β-catenin signaling pathway by targeting *SFRP1*, resulting increased proliferative and invasive abilities (Fig. 4) [59]. Therefore, the three miRNAs mainly display tumor-suppressive functions.

### miR-510-3p and miR-510-5p

As described previously, miR-510-3p and miR-510-5p are upregulated in clinical cancer samples, but their impact on patient prognosis remains uncertain. Functionally, miR-510-3p promotes cancer proliferation and suppresses apoptosis by targeting *PTEN* in non-small cell lung cancer (Fig. 4) [61]. Moreover, miR-510-5p, which can be regulated by lncRNA SNHG15, promotes proliferation, migration, and invasion via targeting *SRCIN1* and *PRDX1* in several cancer cells [60, 62, 96]. Therefore, it is interesting that the two miRNAs are oncogenic even though they have similar sequences to other neighboring miRNAs.

### miR-513 family

There are four precursors and six mature forms of the miR-513 family although their functions are largely unknown (Fig. 1). However, no previous reports have described their 3p strands. In breast cancer cells, miR-513a-5p decreases the expression of *PR*, and it confers resistance to serum starvation stress (Fig. 4) [58]. Hence, miR-513a-5p may be associated with hormone-mediated carcinogenesis. On the other hand, miR-513b-5p overexpression suppresses tumor progression and promotes apoptosis via targeting *HMBG3* (Fig. 2) [45, 46]. Moreover, lncRNA FLVCR1-AS1 promotes cancer progression via sponging miR-513c-5p, which targets *MET* [97]. Therefore, miR-513b-5p and miR-513c-5p are tumor-suppressive.

### miR-514 family

Three precursors of miR-514a synthesize the same mature miRNAs (Fig. 1), and similar to other miRNAs in the cluster, the miR-514 family is involved in the EMT process. In renal cell carcinoma, miR-514a-3p and miR-514a-5p inhibit EMT by targeting *EGFR* and *TRPM3*, respectively (Fig. 2) [47, 98]. Moreover, in lung adenocarcinoma, poor prognostic factors of *TWIST1* and *ZFHX4* are targeted by miR-514a-3p, and *ZFHX4* exerts oncogenic functions by regulating *TWIST1* [99].

The roles of miR-514b-3p and miR-514b-5p are unique and opposite. In colorectal cancer, miR-514b-3p inhibits EMT by targeting *FZD4* and *NTN1*, whereas miR-514b-5p promotes the EMT by targeting *CDH1* and *CLDN1* (Fig. 2&4) [100]. Therefore, the miR-514 family regulates the EMT process.

### miR-888–892 group

The miR-888–892 group is located ~ 1.2 Mb upstream of the miR-506–514 group (Fig. 1); thus, these group have different sequences and functions. The members of the miR-888–892 group are upregulated in the metastatic prostate cancer cells PC3-ML compared with PC3-N [101]. Several reports show that miR-888-5p is oncogenic and promotes the EMT in several cell lines by targeting *SMAD4*, *TIMP2*, and *CDH1* (Fig. 4) [63, 64, 101, 102]. Moreover, *RBL1* and *KLF5* are other target genes of miR-888-5p that might be involved in this function [101]. In hormone-dependent endometrial cancer, miR-888-5p can act as an oncomiR by targeting *PR* [11]. Therefore, miR-888-5p is a key miRNA in the miR-888–892 group. Moreover, miR-891a-5p targets *TIMP2* and may support the functions of miR-888-5p [101]. Furthermore, miR-892a is also oncogenic and targets *PPP2R2A*, a regulator of Akt signaling [65].

This group also contains tumor-suppressive miRNAs. Several reports indicate that miR-892b suppresses proliferation, migration, and invasion via targeting multiple genes such as *CCND1, CDK6*, *LPAR1,* and multiple mediators of NF-kB signaling (Fig. 2) [68, 69, 103]. Moreover, miR-890 and miR-891b inhibit cancer progression by targeting *CD147* and *CBLB*, respectively [67, 71]. Furthermore, miR-890 and miR-891b were shown to sensitize cancer cells to DNA damage by modulating DNA-repair genes [104, 105]. Therefore, miR-890, miR-891b, and miR-892b are tumor-suppressive.

Based on their genome location, the miR-888–892 group can be coexpressed although each miRNA can act both oncogenic and tumor-suppressive. Therefore, the functions of the miR-888–892 group are more cell type- or tissue-specific than those of the miR-506–514 group.

## Future perspectives

The members of the chrXq27.3 miRNA cluster may coordinately regulate cancer-related pathways. The miRNAs in the miR-506–514 group are particularly strong tumor suppressors, and their downregulation plays important roles in cancer progression. Hence, the regulatory mechanisms of this cluster must be elucidated. According to previous reports, miR-506-3p and miR-507 expressions are reduced due to hypermethylation of their promoter region [16, 50]. Conversely, p53 contributes to the increased expressions of miR-506-3p and miR-509-5p [44, 106]. Considering the features of miRNA clusters, other miRNAs in this cluster could be regulated by these factors, and the re-activation of tumor-suppressive miRNAs might be a potent therapeutic strategy. Moreover, the location of the cluster makes itself more interesting because the number of X chromosomes differs between men and women, and it might be responsible for the difference in cancer incidence and immunity between males and females [13].

Additionally, miRNA replacement therapy has been developed, and a phase 1 study about miR-16-based mimic miRNA in malignant pleural mesothelioma was performed [107]. To achieve miRNA replacement therapy in various cancers, a suitable delivery system with a high specificity for targeting cancer cells must be developed. Thus, optimal miRNAs should be selected depending on the cancer types. We believe that the tumor-suppressive miRNAs in this cluster may be suitable because their anti-cancer effect is universal regardless of the cancer. However, the rest of the oncogenic miRNAs in the cluster are also attractive therapeutic targets. Inhibiting the miRNAs may potentially have fewer adverse effects because most of the normal tissues show negligible expression of the miRNAs. Therefore, further studies about this cluster are highly anticipated.

## Conclusion

In conclusion, the chrXq27.3 miRNA cluster is a critical regulator of cancer progression in various types of cancer. Among the 30 mature miRNAs in this cluster, miR-506-3p is the most well-known tumor suppressor, but there are many miRNAs with unknown functions. Therefore, this cluster is worth evaluating in the future and is a promising therapeutic target.

## Data Availability

Not applicable.

## Abbreviations

EMT: epithelial–mesenchymal transition
miRNA: microRNA
SCC: squamous cell carcinoma
